# Sex-Biased Parental Investment among Contemporary Chinese Peasants: Testing the Trivers-Willard Hypothesis

**DOI:** 10.3389/fpsyg.2016.01215

**Published:** 2016-08-17

**Authors:** Liqun Luo, Wei Zhao, Tangmei Weng

**Affiliations:** Department of Sociology, Central China Normal UniversityWuhan, China

**Keywords:** Trivers-Willard hypothesis, natural selection, parental investment, sex ratio, education, Chinese peasants

## Abstract

The Trivers-Willard hypothesis predicts that high-status parents will bias their investment to sons, whereas low-status parents will bias their investment to daughters. Among humans, tests of this hypothesis have yielded mixed results. This study tests the hypothesis using data collected among contemporary peasants in Central South China. We use current family status (rated by our informants) and father's former class identity (assigned by the Chinese Communist Party in the early 1950s) as measures of parental status, and proportion of sons in offspring and offspring's years of education as measures of parental investment. Results show that (i) those families with a higher former class identity such as landlord and rich peasant tend to have a higher socioeconomic status currently, (ii) high-status parents are more likely to have sons than daughters among their biological offspring, and (iii) in higher-status families, the years of education obtained by sons exceed that obtained by daughters to a larger extent than in lower-status families. Thus, the first assumption and the two predictions of the hypothesis are supported by this study. This article contributes a contemporary Chinese case to the testing of the Trivers-Willard hypothesis.

## Introduction

According to the Trivers-Willard hypothesis (TWH), natural selection has shaped the way that parents differentially allocate resources, such as time and energy, to their offspring by sex: parents in good condition during the period of parental investment will invest more in sons, while parents in poor condition during parental investment will invest more in daughters (Trivers and Willard, 1973). The TWH authors hold that the hypothesis is applicable to all sexually reproducing species including humans, and when applied to humans, “good condition” or “bad condition” refers to differentiation on a socioeconomic scale. Parental investment in offspring before birth mainly refers to resource allocation involved in conception and gestation, and is ultimately manifest in sex ratios, body size differences, etc., at birth. On the other hand, parental investment also takes place after the birth of offspring, including food provisioning, lactation, and protection of offspring, etc.

The reasoning of the TWH is based on two assumptions: (i) there is a positive correlation between the condition of parents during parental investment and the condition of offspring at adulthood, and (ii) advantages in condition have greater effects on adult male reproductive success compared to the effects on adult female reproductive success. Thus, following the logic of natural selection, it is not difficult to arrive at two specific predictions of the TWH: as parental condition declines, parents tend to (i) produce a lower sex ratio of males to females at birth, and (ii) allocate relatively more resources to daughters postnatally.

### Tests of the TWH

There is widespread evidence that the two assumptions of the TWH conform to the reality of human societies. For human societies, the first assumption holds that there is relatively stable intergenerational transfers of socioeconomic status. Probably this was most evident in agricultural societies, in which most people's class position was largely determined by family origin and typically remained unchanged for life (Sanderson, 1999). The weakening of social inequality in modern societies has not eliminated the intergenerational transmission of occupational status and wealth at all. Many studies show clear associations between the family of origin and occupation, income or educational attainment, in contemporary Western countries (Erikson and Goldthorpe, 1992; Solon, 2002; Breen, 2004) and other countries such as China (Lin and Bian, 1991; Gong et al., 2012), though they may differ on how the intergenerational transfers vary across countries.

As for the second assumption of the TWH, it implies that higher socioeconomic status should have a greater effect on adult male reproductive success than on that of adult females, and that low-status females' reproductive success should exceed low-status males. The sex difference in reproductive success co-varying with socioeconomic status is strongly supported by data from traditional societies (Betzig, 2012). Moreover, as Hopcroft and Martin (2014, 2016) put it, much recent evidence from the contemporary U.S. and European countries points out that high economic status does promote males', rather than females' reproductive success (Hopcroft, 2005; Fieder and Huber, 2007; Nettle and Pollet, 2008), and lower-income males are less likely to have children (Hopcroft, 2005; Weeden et al., 2006; Barthold et al., 2012). The similar phenomena should take place in other countries such as contemporary China. Much research observes that nearly all women and better educated, more wealthy men marry in China, while the unmarried people mainly consist of low-income and low-education men (Sharygin et al., 2013). In fact, in China, more than 90% of all unmarried people aged 28–49 are men who have not completed high school (Hesketh and Zhu, 2006). As reproduction usually happens within marriages and families, these studies indicate that the reproductive success of low-status Chinese males is lower. Thus the second assumption of the TWH is at least partially supported for China.

Among those tests of the TWH, however, very few examine whether the two assumptions of this hypothesis are consistent to realities of the population studied. An exception is Bereczkei and Dunbar (1997). According to this study, Hungarian Gypsies invest more heavily in daughters than in sons compared to Hungarians who have a relatively higher status. Moreover, the Gypsies have more grandchildren via their daughters, while the data do not show significant differences for the Hungarians. Hence, the second assumption of the TWH is partially vindicated.

As for tests of the two predictions of the TWH in humans, they have yielded mixed results. While historical studies and small-scale studies in contemporary societies tend to support the TWH, large-scale studies in contemporary societies sometimes contradict it, at least its postnatal investment version (Cronk, 2007). The most important reason for those studies not supporting the TWH (e.g., Freese and Powell, 1999; Keller et al., 2001; Beaulieu and Bugental, 2008; Schnettler, 2010) probably comes from problematic measures of family socioeconomic status and postnatal parental investment (Cronk, 2007; Hopcroft and Martin, 2014). These studies use self-reported parental income or education to gauge family status. Self-reports of income may be biased due to the sensitivity of personal income information, and education can roughly, but for understandable reasons, inadequately indicate overall family status. Also, these studies examine postnatal parental investments in offspring at relatively young ages, such as interacting with children. Probably such investments only have a comparatively remote relationship with children's future reproductive success, while the Trivers-Willard effect in certain contemporary societies may only or primarily involve those investments that have a stronger and more direct influence. Like parental income or education for measuring family status, parental investments in children at young ages may not be enough to capture a weak relationship.

To avoid the methodological problems of the above-mentioned studies, Hopcroft (2005) and Hopcroft and Martin (2014, 2016) use children's educational attainment to measure postnatal parental investment, on the basis that children's educational attainment strongly affects their economic, social and reproductive success in contemporary American society. In our view, the effect of education in contemporary Chinese society is similar to that in the United States. In the past decades, income growth in China has been uneven, most rapidly among the educated (Goh et al., 2008). In rural China, education is strongly correlated with both non-farm income and farm income (Knight et al., 2009). In urban China, almost half of the growth in income inequality is due to educational inequality (Zhou, 2009). Education also promotes social prestige. Traditionally a Confucianist country, China has a long history of respecting teachers and the educated, as is manifested in an ancient Chinese saying—a teacher for 1 day is a father for life. Surveys on how residents rate prestige of various occupations in China's Beijing (Lin and Xie, 1988) and Tianjin (Bian, 1996) show that occupations needing more education such as scientists, university teachers, physicians, and engineers, are most valued. As women tend to marry up the socioeconomic scale, education boosting income and prestige will also boost men's reproductive success.

Education also further women's reproductive success. According to Schwartz (2010), a little higher than 50% of American married couples are educationally homogamous, that is, they have similar amounts of education. For Chinese women first married between 1970 and 2001, the percentage of educational homogamy keeps above 49.2 across all six cohorts classified by marriage date, and is even higher than 60 for those married after 1990 (Han, 2010). Since education helps a woman find a more wealthy high-education husband, parental investment in a daughter's education potentially promotes her marital and reproductive success.

Similar to Hopcroft (2005) and Hopcroft and Martin (2014, 2016), this study uses years of education received by children to gauge postnatal parental investment. More years of education mean more investment by parents. Also, different from former studies such as Keller et al. (2001), the present study uses third-party reports to obtain two measures of overall parental status. First, assuming a relatively stable intergenerational transmission of socioeconomic status, we use current family socioeconomic status as a measure of parental status during parental investment. second, as former family class identity reflects family socioeconomic status for quite a long time—before the early 1950s and since the 1980s, we think that including it as a measure of parental status during parental investment besides current family status is justified.

Here, what “previous family class identity” means in the cultural background of China needs to be explained briefly. During the Land Reform by the Chinese Communist Party (CCP) in the late 1940s and early 1950s, each peasant family was assigned one of seven class identities on the basis of family economic status: landlord, rich peasant, upper middle peasant, middle middle peasant, lower middle peasant, poor peasant, and landless laborer. We did field work in the Hunan Province. The Province was taken over by the CCP in 1950 and the land reform there should be conducted in the early 1950s. At that time, some land and other property of landlords and rich peasants were forfeited and redistributed to landless laborers, poor peasants, and lower middle peasants, to effect a more equitable distribution of wealth. Before the Land Reform, landlords and rich peasants were at the top level of the sociopolitical and economic hierarchies in rural China, middle peasants middle, and poor and landless laborers bottom. After the Reform, previous poor and lower-middle peasant families were privileged in many aspects including education and employment, while previous rich and middle class families, especially landlord and rich-peasant families, were disadvantagedly discriminated, until class identities were officially abolished in 1979 (Unger, 1982; Sato and Li, 2008). From the early 1950s to the early 1980s, educational attainment of males was highly egalitarian with respect to social origins (Deng and Treiman, 1997), though evidence about the relationship between social origins and income and women's education is needed. Despite more than two decades of discrimination, children from former landlord, rich-peasant, or middle-peasant families have been more likely to be better educated and to have more family wealth than those from poor or lower-middle peasant families since the 1980s (Sato and Li, 2008, 2013).

### Goals and models

The first goal of this study is to test the first assumption of the TWH, relatively stable intergenerational transmission of socioeconomic status, with data about contemporary Chinese peasants. We expect to find that the higher the family status in the past generations which can be measured by former family class identity, the higher the current family status. In addition, parental age, education, and number of offspring affect current family status. Since the 1980s, China has been experiencing a rapid process of urbanization, during which rural-urban migration is the main source of urban population growth (Chen et al., 2013). Permanent migrants as a whole are young and have higher educational attainments than non-migrants or temporary migrants (Fan, 2008). If we observe older parents and their families in a rural community, we expect that they currently have a lower status there, because some better educated peasants with similar ages to theirs have migrated to cities. As education promotes income, better educated parents and their families should have a higher socioeconomic status. Moreover, sons and daughters can support parents in a variety of ways, e.g., helping manage family business. If we observe parents with more offspring, we also expect that they have a higher status locally.

Second, we wanted to test the first prediction of the TWH, that is, higher-status parents are more likely to have sons than daughters among their biological progeny. We expect that the sex ratios of each peasant family are affected not only by parental status, but also by parental age, education, and number of progeny. First, we assume that older or lower-education parents should be more likely to have been influenced by the traditional son-preference culture and thus to have aborted a female fetus or maltreated their female infants. Other things being equal, the sex ratios at birth for these parents should be higher than others.

Also, number of offspring should affect sex ratio of each family. A coercive birth control policy has been implementing in China since the late 1970s. The basic birth control policy in the rural area studied before 2016 is that a married couple are allowed to have a second child if the first birth is a girl, as is sometimes called “1.5-child” policy (For a review of China's fertility policy at both the local and national levels, see Gu et al., 2007). If a couple only wants a child, son-preference culture might press them to manipulate the sex of the first birth. For a couple having only one or two sons and no daughters, if they cannot afford the fine levied by the government for an unauthorized birth, they would be less likely to have one more child, since they already have had sons. Further, a larger number of offspring means a larger probability of having at least a son. In this case, parents are less likely to manipulate the sex of the next child if they really want one more child. In fact, China's abnormal sex ratios at birth in the past decades (Banister, 2004) is mainly due to manipulation of sex among first and second births (Ebenstein, 2010). All these should cause a negative correlation between number of offspring and sex ratio of each family.

Finally, we wanted to test the second prediction of the TWH that high-status parents invest more in sons postnatally. Similar to the above model, when variables such as parental age, education, and number of offspring are controlled for, we expect that higher-status parents invest relatively more in sons' education, while lower-status parents invest relatively more in daughters'.

## Materials and methods

Prior to data collection, we confirmed with Central China Normal University' s Human Research Ethics Committee that no ethical approval was required for this kind of study, if only participants agreed to participate verbally. We do no report results for any individual participant, but only aggregated results.

### Data collection

Data were collected in July and August 2015 in five adjacent *Han* villages named *Gaotang, Outang, Shuidong, Xianghua*, and *Xiaoling*, respectively, in Shaodong County, central Hunan Province, South Central China. The *Han* Chinese are the majority ethnic group in China, constituting around 90% of the total population. Presently around 50% of the total population in China live in rural areas. According to the information disclosed on the official website of the Shaodong County Government, the gross domestic product (GDP) per capita in Shaodong in 2014 was around 4800 US dollars, while the figure for China as a whole was around 7300 US dollars in the same year, in terms of the present USD to RMB exchange rate.

To collect the data, we first copied the pages of household registers about family heads from the police department. Under China's household registration system, usually every family register in a local police department the basic information about each family member' sex, birth date, ethnicity, etc. But the registers do not necessarily perfectly reflect the birth, death, and migration of a local population. For example, those children who were born with no official approval and whose parents were unable to pay the fines levied by the government usually are excluded from the registration system. The registers also do not cover the information about family members' current socioeconomic status and previous family class identity, which is crucial for this study.

As the registers provided the information about each famiy head's sex and age, then we tried to find two informants in each village to obtain other relevant information about each family. One informant was a retired older-than-60 village head, Chinese Communist Party (CCP) branch secretary in the village, or village accountant. The other was the current village head, CCP branch secretary in the village, village accountant, or a teacher of the local primary school, with a middle age. We paid each of them about 32 US dollars as reward in advance. They informed us of each family head's education level, current family socioeconomic status in the local community, fathers' previous class identity, and each biological son's and daughter's birth year and education level. All the informants kept living in the investigated area for decades. In every village, usually both informants easily concurred on the above information.

The reason that we did not directly interview the family heads for primary data was that many peasants were oversensitive to questions about offspring, current family status, and father's former class identity. In our preliminary fieldwork conducted in June 2015, an interviewed peasant with a landlord family origin told us that his family origin was poor peasant, presumably because his father was persecuted to death during Chairman Mao's era only for being a previous landlord and this event connected to class identity was a trauma for him. Like many Western people, some Chinese peasants regard personal or familial socioeconomic status as privacy. Moreover, many peasant parents have one or more unauthorized births. They would not like strangers to know this, lest this would be reported to the local government.

We finally obtained intact information about 1418 family heads (1241 males and 177 females). See Table 1 below.

**Table 1:** **Descriptive statistics for family heads**.

**Variable**	**Mean**	***SD***	**Min**	**Max**
Sex (Male = 1, *N* = 1418)	0.88	0.33	0	1
Age (*N* = 1418)	59.87	12.83	25	97
Years of education (*N* = 1418)	7.60	2.90	0.00	16.00
Number of offspring (*N* = 1418)	2.27	1.10	0.00	7
Current family socioeconomic status (*N* = 1418)	2.09	0.64	1	3
Former class identity (*N* = 1418)	1.13	0.42	1	3
Proportion of sons in offspring (*N* = 1368)	0.59	0.31	0.00	1.00
Ratio of sons' average education to daughters' (*N* = 674)^a^	1.13	0.31	0.00	4.50
Offspring sex (Male = 1, *N* = 2818)^b^	0.55	0.50	0	1
Offspring's years of education (*N* = 2818)^b^	10.40	2.79	0.00	22.00

a *The variable ratio of sons' average education to daughters'in this table and Table 5 only involves those family heads who have at least a son and a daughter and whose offspring are at least 18 years old*.

b *The variables offspring sex and offspring's years of education in this table and Table 4 only involve those offspring who are at least 18 years of age*.

### Outcome variables

Testing the first assumption of the TWH, we used *current family socioeconomic status* as the outcome variable. A score of 1 means bad family socioeconomic status in the local community, a score of 2 means average status, and 3 means good status.

In our pilot fieldwork in January 2015, we discovered that Chinese peasants usually classified their general family socioeconomic status into three categories of “bad,” “average,” and “good.” More specific classifications seemed to confound them sometimes. It is this trichotomy that we finally used to collect the data presented here.

In testing the first prediction of the TWH, we used *proportion of sons in offspring* as the outcome variable. We divided the number of sons of each family head by the total number of his/her offspring to obtain this variable, for all those with at least a child. For example, if a family head has a son and a daughter, he or she will obtain a score of 0.5 on the variable. The greater the value, the relatively more prenatal investment in sons.

As for testing of the second prediction of the TWH, we used *offspring's years of education* as an outcome variable, for all the 2818 offspring (in the 1230 families) who were at least 18 years of age. Also, for the 674 family heads who had at least a son and a daughter and all of whose offspring were at least 18 years old, we divided the average years of education of sons by the average years of education of daughters to obtain a new variable, *ratio of sons' average education to daughters'*, for gauging sex-biased postnatal parental investment. For example, for a family head, if two sons' years of education are 6 and 9 years, and two daughters' years of education are 6 and 16, then the average years of education for sons and for daughters will be 7.5 and 11, and the variable *ratio of sons' average education to daughters'* will have a score of 7.5/11=0.68. The greater the value, the relatively more postnatal parental investment in sons.

### Independent variables

In testing the first assumption of the TWH, we used each family head's *age, years of education, number of offspring*, and father's *former class identity* as independent variables. As *current family socioeconomic status* is an ordinal variable and the test of parallel lines showed that the data did not meet the proportional odds assumption for performing an ordinal logit regression analysis (Norusis, 2012), the above variables were entered into an ordinal probit regression.

The variable *former class identity* indicates each family head's father's previous class identity. In the Chinese context, a person's father's or paternal grandfather's former class identity is also regarded as the person's previous family class identity. For the variable, score of 1, 2, or 3 mean that the previous class identity is poor peasant or landless laborer, middle class peasant, or landlord or rich peasant, respectively. The three kinds of class identities correspond to good-class origins, middle-class origins, and bad-class origins from the 1950s to the late 1970s in Maoist China (Unger, 1982; Deng and Treiman, 1997).

In testing the first prediction of the TWH, we used each family head's *age, years of education, current family socioeconomic status*, and *former class identity* as independent variables. Considering that the dependent variable had only a few values and that the proportional odds assumption was not met, we performed an ordinal probit regression of the dependent variable.

In testing the second prediction of the TWH, we performed two multilevel regressions of *offspring's year of education*. In both models, family head's *age, years of education*, and *number of offspring* were used to estimate random effects. *Offspring's sex* was coded as Male = 1, Female = 0. In model 1, *offspring's sex*, each family head's *current family socioeconomic status* and *former class identity*, and offspring's sex^*^ *current family socioeconomic status*, were used to estimate fixed effects. On the other hand, *offspring's sex*, each family head's *current family socioeconomic status* and former class identity, and offspring's sex^*^ *former class identity*, were included in model 2 to estimate fixed effects.

All data analyses were carried out using STATA. All tests were one-tailed unless otherwise specified.

## Results

Table 1 presents the descriptive statistics for all variables used in the analysis. Zero-order Pearson correlations are given in the Appendix.

### Testing the first assumption of the TWH

As expected, all the four independent variables are significant predictors of the dependent variable current family socioeconomic status (Table 2). The value of tolerance for each independent variable is much greater than the 0.10 collinearity threshold. This means that despite the correlations between the predictor variables the independent effect of each variable on the dependent variable was statistically confirmed. This model shows that younger, better educated family heads who have more offspring and whose family's socioeconomic status before the early 1950s was higher, tend to have a higher family socioeconomic status currently. As far as the first assumption of the TWH is concerned, the model shows that when the other three independent variables are controlled, former class identity has an independent, positive influence on current family status. The effect size (Spearman rank correlation coefficient *r*_*s*_) between former class identity and current family socioeconomic status is 0.079 (*n* = 1418, *p* = 0.001).

**Table 2:** **Ordered probit regression of current family socioeconomic status on independent variables**.

**Independent variables**	**Coef**.	**SE**	***p***	**Tolerance^a^**
Age	−0.006	0.003	0.029	0.519
Years of education	0.129	0.014	0.000	0.616
Number of offspring	0.167	0.032	0.000	0.760
Former class identity	0.129	0.073	0.039	0.980

*N = 1418, Pseudo R^2^ = 0.062*.

a *The values of tolerance in Tables 2 and 3 were obtained from the Ordinary Least Squares regression using the same dependent and independent variables (Menard, 2002)*.

Another interesting finding is concerned with the relationship between number of offspring and family status. The Chinese government has been propagandizing for decades “the more children, the poorer,” to urge people to control birth. Ironically, when number of offspring was assumed to really influence subsequent socioeconomic status and was entered into the above regression, we found that it could enhance, rather than weaken family status.

### Testing the first prediction of the TWH

Results of the test of prenatal investment in offspring (Table 3) show that all the independent variables except years of education are statistically significant. Similar to the first regression analysis above, the value of tolerance for each independent variable is much greater than the 0.10 collinearity threshold. Directions of the relationships in the model are consistent with our expectation—a family head with an older age, fewer offspring, and higher current family status and former class identity, is more likely to have sons.

**Table 3:** **Ordered probit regression of proportion of sons in offspring on independent variables**.

**Independent variables**	**Coef**.	**SE**	***p***	**Tolerance**
Age	0.014	0.003	0.000	0.486
Years of education	−0.014	0.014	0.144	0.582
Number of offspring	−0.358	0.036	0.000	0.644
Current family socioeconomic status	0.138	0.049	0.003	0.918
Former class identity	0.152	0.070	0.015	0.979

*N = 1368, Pseudo R^2^ = 0.024*.

As the value of current family socioeconomic status and former class identity rises, the mean value of proportion of sons in offspring keeps rising (**Table 5**). The effect sizes (Spearman *r*_*s*_) for current family socioeconomic status and former class identity in predicting proportion of sons in offspring are 0.052 (*n* = 1368, *p* = 0.028) and 0.042 (*n* = 1368, *p* = 0.060), respectively. To sum up, the first prediction of the TWH is confirmed.

### Testing the second prediction of the TWH

Results of the test of postnatal investment in offspring (Table 4) show that for family heads with a higher current family status, their offspring attain more years of education than others. Contrary to our expectation, in model 2, former class identity is negatively associated with offspring's years of education. We suspect that this is due to that some better educated peasants with a family origin of landlord, rich peasant or middle peasant have moved out to cities. This model also shows significant interaction terms of offspring sex by current family status or previous family class identity in the positive direction predicted by the TWH—An increase in current family status or former family class identity produces a greater increase in the education of sons than of daughters.

**Table 4:** **Multilevel regression of offspring's years of education on independent variables: fixed-effect parameters^a^**.

**Independent variables**	**Model 1**		**Model 2**	
	**Coef**.	***p***	**Coef**.	***p***
Intercept	8.552 (0.323)	0.000	8.613 (0.314)	0.000
Offspring's sex (Male=1)	0.239 (0.272)	0.379	0.190 (0.221)	0.389
Current family socioeconomic status	0.927 (0.128)	0.000	1.090 (0.106)	0.000
Former class identity	−0.206 (0.157)	0.188	−0.554 (0.192)	0.004
Offspring's sex^*^current family socioeconomic status	0.282 (0.125)	0.024		
Offspring's sex^*^former class identity			0.553 (0.179)	0.002

*Standard errors in parentheses*.

*Number of offspring = 2818, number of families = 1230, two-tailed tests*.

a *The inclusion of the interaction terms in the model causes collinearity to some extent. Because collinearity in this case can be safely ignored (Friedrich, 1982), we do not report tolerance values here*.

The mean value of ratio of sons' average education to daughters' in each family goes up hand in hand with current family socioeconomic status and former class identity (Table 5). The trends are consistent and stable, though the changes are small. The effect sizes (Spearman *r*_*s*_) for current family socioeconomic status and former class identity in predicting ratio of sons' average education to daughters' are 0.077 (*n* = 674, *p* = 0.023) and 0.081 (*n* = 674, *p* = 0.018), respectively. To conclude, the second prediction of the TWH is supported.

**Table 5:** **Means of proportion of sons in offspring and means of ratio of sons' average education to daughters' by family status**.

	**Mean of proportion of sons in offspring**	**Mean of ratio of sons' average education to daughters'**
Current family socioeconomic Status = 1^a^	0.544 (*N* = 195)	1.114 (*N* = 103)
Current family socioeconomic status = 2	0.588 (*N* = 824)	1.117 (*N* = 396)
Current family socioeconomic status = 3	0.613 (*N* = 349)	1.180 (*N* = 175)
Former class identity = 1^b^	0.584 (*N* = 1232)	1.124 (*N* = 597)
Former class identity = 2	0.600 (*N* = 91)	1.181 (*N* = 55)
Former class identity = 3	0.672 (*N* = 45)	1.242 (*n* = 22)

a *For the variable current family socioeconomic status, a score of 1 means bad family socioeconomic status in the local community, a score of 2 means average status in the local community, and 3 means good status*.

b *For the variable former class identity, a score of 1 means that the previous family class identity is poor peasant or landless laborer, a score of 2 means that the previous family class identity is middle peasant, and a score of 3 means landlord or rich peasant*.

## Discussion

Taken together, the data from this study support the validity of the first assumption and the two predictions of the TWH among contemporary Chinese peasants. First, we found that those family heads whose father had a higher class identity assigned by the CCP in the early 1950s tended to have a family with a higher socioeconomic status currently. This means that there were transfers of socioeconomic status across generations to some extent in the previous decades. Thus the first assumption of the TWH is supported. Probably this is the first time for testing that assumption in a population studied. Second, when parental socioeconomic status during parental investment was measured by both current status and former class identity, we found that high-status parents were more likely to have sons than daughters among their biological offspring. Finally, the data show that as family heads' current family status or former class identity increases, the education of sons increases to a larger extent than that of daughters. This means that high-status parents invest relatively more in the education of sons compared to low-status parents.

In humans, the TWH is essentially involved with the relationships between parental socioeconomic status and sex-biased parental investment in children. Similar to several earlier studies (e.g., Bereczkei and Dunbar, 1997; Cox, 2003; Hopcroft, 2005; Pollet et al., 2009; Wallner et al., 2012; Hopcroft and Martin, 2014, 2016), this study based the measure of parental status on overall status, and the measure of parental investment on investment in the education of offspring. All the studies obtained positive results regarding the TWH, in contrast to some other studies (e.g., Freese and Powell, 1999; Keller et al., 2001; Beaulieu and Bugental, 2008; Schnettler, 2010) that used different measures. As the TWH only indicates a weak effect, the usage of more proper measures of parental status, and parental investment such as educational investment that is more important for children's future reproductive success, should have been more suited for testing this hypothesis.

Along with previous studies (Bereczkei and Dunbar, 1997; Cox, 2003; Hopcroft, 2005; Hopcroft and Martin, 2014, 2016), this study shows that on average the educational benefits of having high-status parents is greater for sons than for daughters. Furthermore, the present study used the variable *ratio of sons' average education to daughters* to examine sex difference in educational investment in offspring within each family. In fact, we found that the mean value of this variable kept rising as family status increased.

Also, similar to such studies supporting the TWH as Voland et al. (1997) and Cronk (2000), this study focuses on a social class (peasants), rather than the whole population of a nation. In our view, a social class (e.g., peasants or manual workers) seems to be more similar to a population in a biological sense than a whole nation. More specifically, first, studies show that people tend to marry within their social group or marry someone who is close to them in status (Kalmijn, 1998). Second, members of a social class should be more likely to live in similar or even the same residential areas. Third, human social behaviors should occur within each social class to a very large degree. If a social class such as peasants is differentiated within itself on a socioeconomic scale and meets the two assumption of the TWH, the TWH should be applicable. We expect that future human studies testing the TWH in such social classes will lend more support to the hypothesis that was developed from a biological perspective, though variation between different social classes in parental investment might sometimes cause the TWH inapplicable to a whole nation.

But some people may complain that cultural factors are ignored in the testing of the TWH. In fact, many studies (e.g., Li and Cooney, 1993; Gupta et al., 2003) insist that East and South Asian countries such as China have a deep-rooted, universal cultural norm of son preference. This may raise the question that whether or not the TWH is applicable in these regions. In our view, however, the problem of son preference has been greatly exaggerated. We have never seen any quantitative study to convincingly prove that almost everyone in these countries, regardless of social class or socioeconomic status, prefers sons to daughters. As we mentioned above, the TWH can apply to every social class that is differentiated on a socioeconomic scale. In this case, probably son preference would have evolved out first in high status individuals in each social class. According to the theory of cultural evolution developed mainly by anthropologists and biologists, there are two key social learning biases, that is, payoff bias (individuals tend to learn from other individuals who have succeeded to some extent in terms of higher payoffs) (Boyd and Richerson, 1985) and conformist bias (individuals tend to learn the most common behaviors in a population) (Henrich and Boyd, 1998). If these two biases hold, it is not surprising that many or even most individuals in a country such as China have a cultural norm of son preference. Compared to natural selection, however, culture itself is only a minor force for molding human behavior. Moreover, organisms including humans often deceive themselves and humans do not necessarily follow what they have said about themselves or their cultural norms (Trivers, 2011). To summarize, that many individuals hold a cultural norm of son preference does not contradict the TWH. As long as the two assumptions of the TWH hold, son preference and the consequent distorted sex ratio will not change the predictions of the hypothesis fundamentally.

The TWH provides an evolutionary or ultimate explanation for sex-biased parental investment, concerned with the fitness consequences of parental behaviors. The present study discovers what the TWH predicts in contemporary Chinese peasants. However, the specific mechanisms by which the results come about are unclear. For example, peasants' choice of educational investment in offspring may be directly influenced by schooling costs or manual labor needs. As ultimate and mechanistic or proximate explanations are complementary, future studies that clarify the mechanisms will help us achieve a fuller understanding of the Trivers-Willard effect.

## Author contributions

LL designed the study. WZ and TW collected the data. All of the authors discussed the data and wrote the paper.

### Conflict of interest statement

The authors declare that the research was conducted in the absence of any commercial or financial relationships that could be construed as a potential conflict of interest.

## Appendix

**Table A1:** **Pearson correlations between all variables regarding each family head**.

**Variable**	**Sex (Male = 1)**	**Age**	**Years of education**	**Number of offspring**	**Current Family SES**	**Former class identity**	**Proportion of sons in offspring**	**Ratio of sons' average education to daughters'**
Sex (Male = 1)	1							
Age	−0.194^***^ (*n* = 1418)	1						
Years of education	0.224^***^ (*n* = 1418)	−0.612^***^ (*n* = 1418)	1					
Number of offspring	−0.201^***^ (*n* = 1418)	0.489^***^ (*n* = 1418)	−0.302^***^ (*n* = 1418)	1				
Current Family Socioeconomic status	0.000 (*n* = 1418)	−0.178^***^ (*n* = 1418)	0.304^***^ (*n* = 1418)	0.027 (*n* = 1418)	1			
Former class identity	−0.041^†^ (*n* = 1418)	0.056^*^ (*n* = 1418)	0.063^**^ (*n* = 1418)	0.063^**^ (*n* = 1418)	0.069^**^ (*n* = 1418)	1		
Proportion of sons in offspring	−0.017 (*n* = 1368)	−0.026 (*n* = 1368)	0.033 (*n* = 1368)	−0.223^***^ (*n* = 1368)	0.066^**^ (*n* = 1368)	0.047^*^ (*n* = 1368)	1	
Ratio of sons' average education to daughters'	−0.001 (*n* = 674)	0.131^***^ (*n* = 674)	−0.075^*^ (*n* = 674)	0.123^**^ (*n* = 674)	0.078^*^ (*n* = 674)	0.082^*^ (*n* = 674)	0.037 (*n* = 674)	1

† p < 0.10;

* p < 0.05;

** p < 0.01;

*** *p < 0.001*.

## References

[B1] Banister J. (2004). Shortage of girls in China today. J. Popul. Res. 21, 19–45. 10.1007/BF03032209

[B2] Barthold J. A. Myrskylä M. Jones O. R. (2012). Childlessness drives the sex difference in the association between income and reproductive success of modern Europeans. Evol. Hum. Behav. 33, 628–638. 10.1016/j.evolhumbehav.2012.03.003

[B3] Beaulieu D. A. Bugental D. (2008). Contingent parental investment: an evolutionary framework for understanding early interaction between mothers and children. Evol. Hum. Behav. 29, 249–255. 10.1016/j.evolhumbehav.2008.01.002

[B4] Bereczkei T. Dunbar R. I. M. (1997). Female-biased reproductive strategies in a Hungarian Gypsy population. Proc. R. Soc. B 264, 17–22. 10.1098/rspb.1997.0003

[B5] Betzig L. (2012). Means, variances, and ranges in reproductive success: comparative evidence. Evol. Hum. Behav. 33, 309–317. 10.1016/j.evolhumbehav.2011.10.008

[B6] Bian Y. (1996). Chinese occupational prestige: a comparative analysis. Int. Sociol. 11, 161–186. 10.1177/026858096011002002

[B7] Boyd R. Richerson P. J. (1985). Culture and the Evolutionary Process. Chicago, IL: University of Chicago Press.

[B8] Breen R. (2004). Social Mobility in Europe. Oxford, UK: Oxford University Press.

[B9] Chen M. Liu W. Tao X. (2013). Evolution and assessment on China's urbanization 1960–2010: under-urbanization or over-urbanization? Habitat Int. 38, 25–33. 10.1016/j.habitatint.2012.09.00712266026

[B10] Cox D. (2003). Private transfers within the family: mothers, fathers, sons, and daughters, in Death and Dollars: The Role of Gifts and Bequests in America, eds Munnell A. Sundén A. (Washington, DC: Brookings Institution Press), 168–217.

[B11] Cronk L. (2000). Female-biased parental investment and growth performance among the Mukogodo, in Adaptation and Human Behavior: An Anthropological Perspective, eds Cronk L. Chagnon N. Irons W. (New York, NY: Aldine De Gruyter), 203–221.

[B12] Cronk L. (2007). Boy or girl: gender preferences from a Darwinian point of view. Reprod. Biomed. Online 15, 23–32. 10.1016/S1472-6483(10)60546-9 18088517

[B13] Deng Z. Treiman D. J. (1997). The impact of the cultural revolution on trends in educational attainment in the People's Republic of China. Am. J. Sociol. 103, 391–428. 10.1086/231212

[B14] Ebenstein A. (2010). The “missing girls” of China and the unintended consequences of the one child policy. J. Hum. Resour. 45, 87–115. 10.3368/jhr.45.1.87

[B15] Erikson R. Goldthorpe J. H. (1992). The Constant Flux: A Study of Class Mobility in Industrial Societies. Oxford, UK: Clarendon.

[B16] Fan C. C. (2008). China on the Move: Migration, the State, and the Household. New York, NY: Routledge.

[B17] Fieder M. Huber S. (2007). The effects of sex and childlessness on the association between status and reproductive output in modern society. Evol. Hum. Behav. 28, 392–398. 10.1016/j.evolhumbehav.2007.05.004

[B18] Freese J. Powell B. (1999). Sociobiology, status and parental investment in sons and daughters: testing the Trivers-Willard hypothesis. Am. J. Sociol. 104, 1704–1743. 10.1086/210221

[B19] Friedrich R. J. (1982). In defense of multiplicative terms in multiple regression equations. Am. J. Polit. Sci. 26, 797–833. 10.2307/2110973

[B20] Goh C. Luo X. Zhu N. (2008). Income growth, inequality and poverty reduction: a case study of eight provinces in China. China Econ. Rev. 20, 485–496. 10.1016/j.chieco.2008.10.008

[B21] Gong H. Leigh A. Meng X. (2012). Intergenerational income mobility in urban China. Rev. Income Wealth 58, 481–503. 10.1111/j.1475-4991.2012.00495.x

[B22] Gu B. Wang F. Guo Z. Zhang E. (2007). China's local and national fertility policies at the end of the twentieth century. Popul. Dev. Rev. 33, 129–147. 10.1111/j.1728-4457.2007.00161.x

[B23] Gupta M. D. Zhenghua J. Bohua L. Zhenming X. Chung W. Hwa-Ok B. (2003). Why is son preference so persistent in East and South Asia? Across-country study of China, India and the Republic of Korea. J. Dev. Stud. 40, 153–187. 10.1080/00220380412331293807

[B24] Han H. (2010). Trends in educational assortative marriage in China from 1970 to 2000. Demogr. Res. 22, 733–770. 10.4054/DemRes.2010.22.24

[B25] Henrich J. Boyd R. (1998). The evolution of conformist transmission and the emergence of between-group differences. Evol. Hum. Behav. 19, 215–241. 10.1016/S1090-5138(98)00018-X

[B26] Hesketh T. Zhu W. X. (2006). Abnormal sex ratios in human populations: causes and consequences. Proc. Natl. Acad. Sci. U.S.A. 103, 13271–13275. 10.1073/pnas.0602203103 16938885 PMC1569153

[B27] Hopcroft R. L. (2005). Parental status and differential investment in sons and daughters: Trivers-Willard revisited. Soc. Forces 83, 1111–1136. 10.1353/sof.2005.0035

[B28] Hopcroft R. L. Martin D. O. (2014). The primary parental investment in children in the contemporary USA is education: testing the Trivers-Willard hypothesis of parental investment. Hum. Nat. 25, 235–250. 10.1007/s12110-014-9197-0 24610663

[B29] Hopcroft R. L. Martin D. O. (2016). Parental investments and educational outcomes: Trivers–Willard in the US. Front. Sociol. 1:3 10.3389/fsoc.2016.00003

[B30] Kalmijn M. (1998). Intermarriage and homogamy: causes, patterns, trends. Annu. Rev. Sociol. 24, 395–421. 10.1146/annurev.soc.24.1.395 12321971

[B31] Keller M. C. Nesse R. M. Hofferth S. (2001). The Trivers-Willard hypothesis of parental investment. No effect in the contemporary United States. Evol. Hum. Behav. 22, 343–360. 10.1016/S1090-5138(01)00075-7

[B32] Knight J. Li S. Deng Q. (2009). Education and the poverty trap in rural China: setting the trap. Oxford Dev. Stud. 37, 311–332. 10.1080/13600810903305232

[B33] Li J. Cooney R. S. (1993). Son preference and the one child policy in China: 1979–1988. Popul. Res. Policy Rev. 12, 277–296. 10.1007/BF01074389

[B34] Lin N. Bian Y. (1991). Getting ahead in urban China. Am. J. Sociol. 97, 657–88. 10.1086/229816

[B35] Lin N. Xie W. (1988). Occupational prestige in urban China. Am. J. Sociol. 93, 793–832. 10.1086/228825

[B36] Menard S. W. (2002). Applied Logistic Regression Analysis. Thousand Oaks, CA: Sage 10.4135/9781412983433

[B37] Nettle D. Pollet T. V. (2008). Natural selection on male wealth in humans. Am. Nat. 172, 658–666. 10.1086/591690 18808363

[B38] Norusis M. J. (2012). IBM SPSS Statistics 19 Advanced Statistical Procedures Companion. Upper Saddle River, NJ: Prentice Hall.

[B39] Pollet T. V. Fawcett T. W. Buunk A. P. Nettle D. (2009). Sex-ratio biasing towards daughters among lower-ranking co-wives in Rwanda. Biol. Lett. 5, 765–768. 10.1098/rsbl.2009.0394 19586966 PMC2827982

[B40] Sanderson S. (1999). Macrosociology: An Introduction to Human Societies, 4th Edn. New York, NY: Longman.

[B41] Sato H. Li S. (2008). Class origin, family culture, and education in rural China (in Chinese). China Econ. Q. 7, 1105–1129. 10.13821/j.cnki.ceq.2008.04.003

[B42] Sato H. Li S. (2013). Influence of family background on current family wealth in rural China. Hitotsub. J. Econ. 54, 95–117.

[B43] Schnettler S. (2010). Nature Plus Nurture Equals Love? A Test of the Trivers-Willard Hypothesis of Differential Parental Investment on the Basis of Sociological and Biological Explanations. Dissertation. New Haven, CT: Yale University.

[B44] Schwartz C. R. (2010). Pathways to educational homogamy in marital and cohabiting unions. Demography 47, 735–753. 10.1353/dem.0.0124 20879686 PMC3000057

[B45] Sharygin E. Ebenstein A. Das Gupta M. (2013). Implications of China's future bride shortage for the geographical distribution and social protection needs of never-married men. Pop. Stud. 67, 39–59. 10.1080/00324728.2012.723893 23227821 PMC3997210

[B46] Solon G. (2002). Cross-country differences in intergenerational earnings mobility. J. Econ. Perspect. 16, 59–66. 10.1257/089533002760278712

[B47] Trivers R. (2011). The Folly of Fools: The Logic of Deceit and Self-Deception in Human Life. New York, NY: Basic Books.

[B48] Trivers R. L. Willard D. E. (1973). Natural selection of parental ability to vary the sex ratio of offspring. Science 179, 90–92. 10.1126/science.179.4068.90 4682135

[B49] Unger J. (1982). Education under Mao: Class and Competition in Canton Schools, 1960–1980. New York, NY: Columbia University Press.

[B50] Voland E. Dunbar R. I. M. Engel C. Stephan P. (1997). Population increase and sex-biased parental investment in humans: evidence from 18th-and 19th-century Germany. Curr. Anthropol. 38, 129–135. 10.1086/204593

[B51] Wallner B. Fieder M. Seidler H. (2012). Ownership of dwelling affects the sex ratio at birth in Uganda. PLoS ONE 7:e51463. 10.1371/journal.pone.0051463 23284697 PMC3524175

[B52] Weeden J. Abrams M. J. Green M. C. Sabini J. (2006). Do high-status people really have fewer children? Education, income, and fertility in the contemporary U.S. Hum. Nat. 17, 377–392. 10.1007/s12110-006-1001-3 26181608

[B53] Zhou X. (2009). Increasing returns to education, changing labor force structure, and the rise of earnings inequality in urban China, 1996–2010. Soc. Forces 93, 429–455. 10.1093/sf/sou073

